# Adsorption of anionic surfactant at the electrode–NaClO_4_ solution interface

**DOI:** 10.1007/s00706-014-1382-7

**Published:** 2015-01-21

**Authors:** Dorota Gugała-Fekner, Jolanta Nieszporek, Dorota Sieńko

**Affiliations:** Faculty of Chemistry, M.Curie-Sklodowska University, Lublin, Poland

**Keywords:** Differential capacity, Adsorption isotherms, Anionic surfactant, Electrosorption, Mercury electrode

## Abstract

**Abstract:**

Adsorption of 1-decanesulfonic acid at the electrode–NaClO_4_ solution interface was determined by double-layer differential capacity measurements. At potentials less than −1,200 mV, the adsorption of the anionic surfactant on the electrode does not occur. Low concentrations of the anionic surfactant (below cmc) causes slight changes in the zero charge potential, *E*_z_, and the surface tension at this potential, *γ*_z_. The adsorption of the anionic surfactant was analyzed using the constants obtained from the following isotherms: Frumkin, corrected Flory–Huggins, and virial.

**Graphical Abstract:**

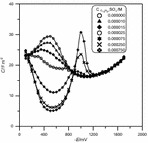

## Introduction

Adsorption of ionic surfactants on the electrode is a topic of both practical and academic interest. Ionic surfactants have been applied in many fields, e.g. water purification, toiletry confection, oil exploitation, etc. [1]. Anionic surfactants are amphipathic compounds consisting of a hydrophobic part, e.g. alkyl chain of various lengths, and a hydrophilic part, e.g. a sulfonate. It has been established many times that the hydrophobic and hydrophilic parts readily interact with polar and non-polar molecules in a mixture of compounds [2, 3]. Adsorption of anionic surfactants on interfaces [4, 5] can modify surface characteristics and electron transfer [6].

In most cases, the application of surfactants is based on empirical knowledge. However, new technologies require basic knowledge about the mode of operation of surfactants and their adsorption mechanisms.

The aim of this work is to study the adsorption processes of 1-decanesulfonic acid anion ${\text{C}}_{ 1 0} {\text{H}}_{ 2 1} {\text{SO}}_{ 3}^{ - }$ at the mercury electrode. The homogeneity and purity of the surface of mercury provide excellent reproducibility of adsorption phenomena. Our choice of NaClO_4_ as the supporting electrolyte results from the fact that ${\text{ClO}}_{ 4}^{ - }$ ions cause the strongest disruption of water structure [7]. In experimental studies on the adsorption from the solution, the double-layer capacitance was usually chosen as the primary experimental quantity. The chosen surfactant concentrations are lower than their critical micellar concentration (cmc).

## Results and discussion

### Analysis of experimental data

**Fig. 1:**
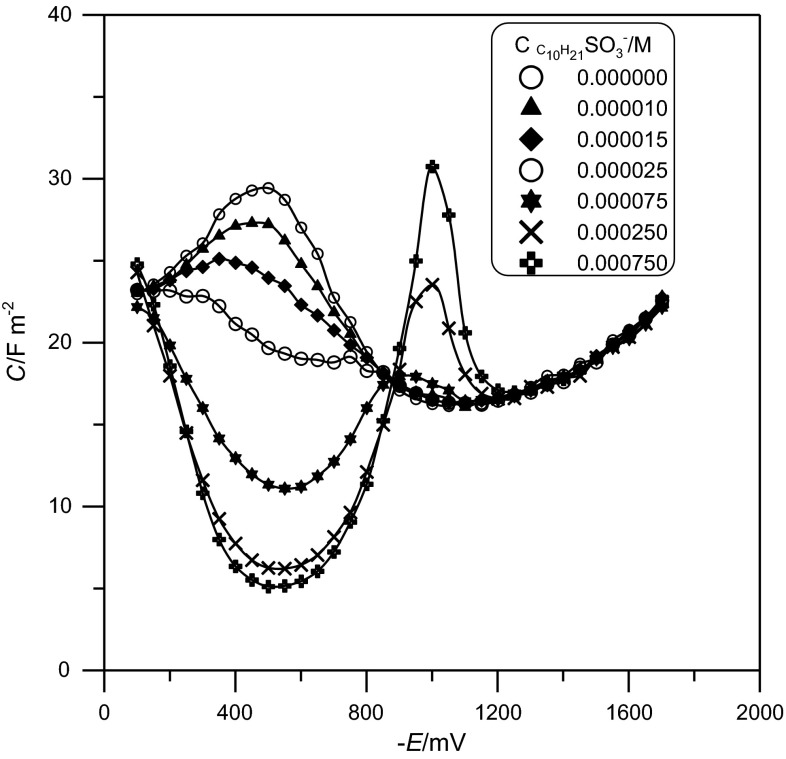
Differential capacity–potential curves of Hg/1 M NaClO_4_ and various ${\text{C}}_{ 1 0} {\text{H}}_{ 2 1} {\text{SO}}_{ 3}^{ - }$ concentrations, as in the figure legend

Figure 1 presents differential capacity curves obtained experimentally in 1 M NaClO_4_ (a) and in a chosen ${\text{C}}_{ 1 0} {\text{H}}_{ 2 1} {\text{SO}}_{ 3}^{ - }$ concentration. By introducing ${\text{C}}_{ 1 0} {\text{H}}_{ 2 1} {\text{SO}}_{ 3}^{ - }$ to the supporting electrolyte a distinct decrease in differential capacity was caused from −100 to −900 mV. At higher ${\text{C}}_{ 1 0} {\text{H}}_{ 2 1} {\text{SO}}_{ 3}^{ - }$ concentrations, a desorption peak is obtained at −1,000 mV. At potentials less than −1,200 mV, the adsorption of the anionic surfactant on the electrode does not occur.

**Fig. 2:**
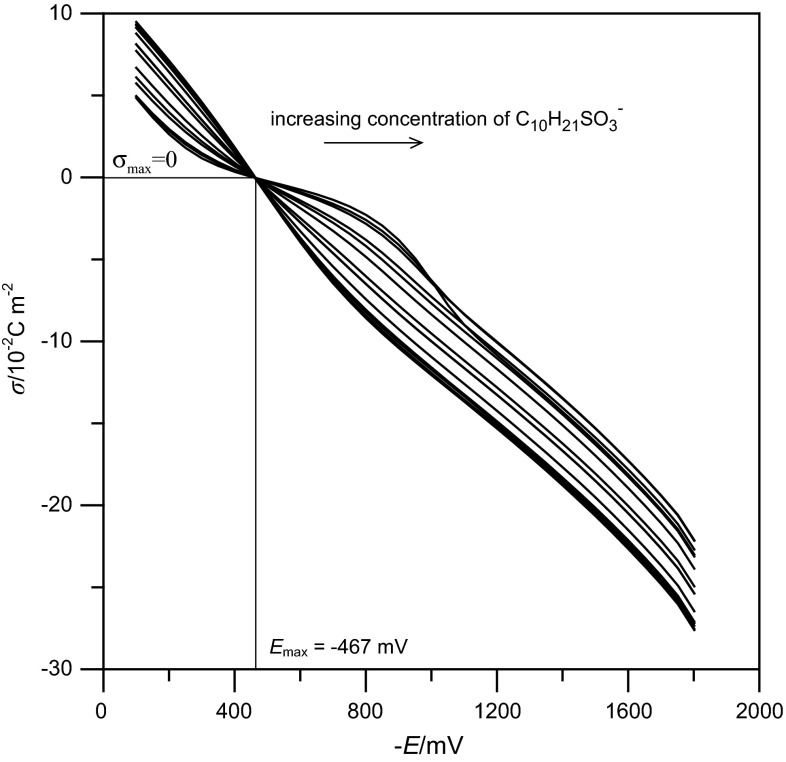
Dependence of the electrode charge on the electrode potential for the studied ${\text{C}}_{ 1 0} {\text{H}}_{ 2 1} {\text{SO}}_{ 3}^{ - }$ concentrations

**Fig. 3:**
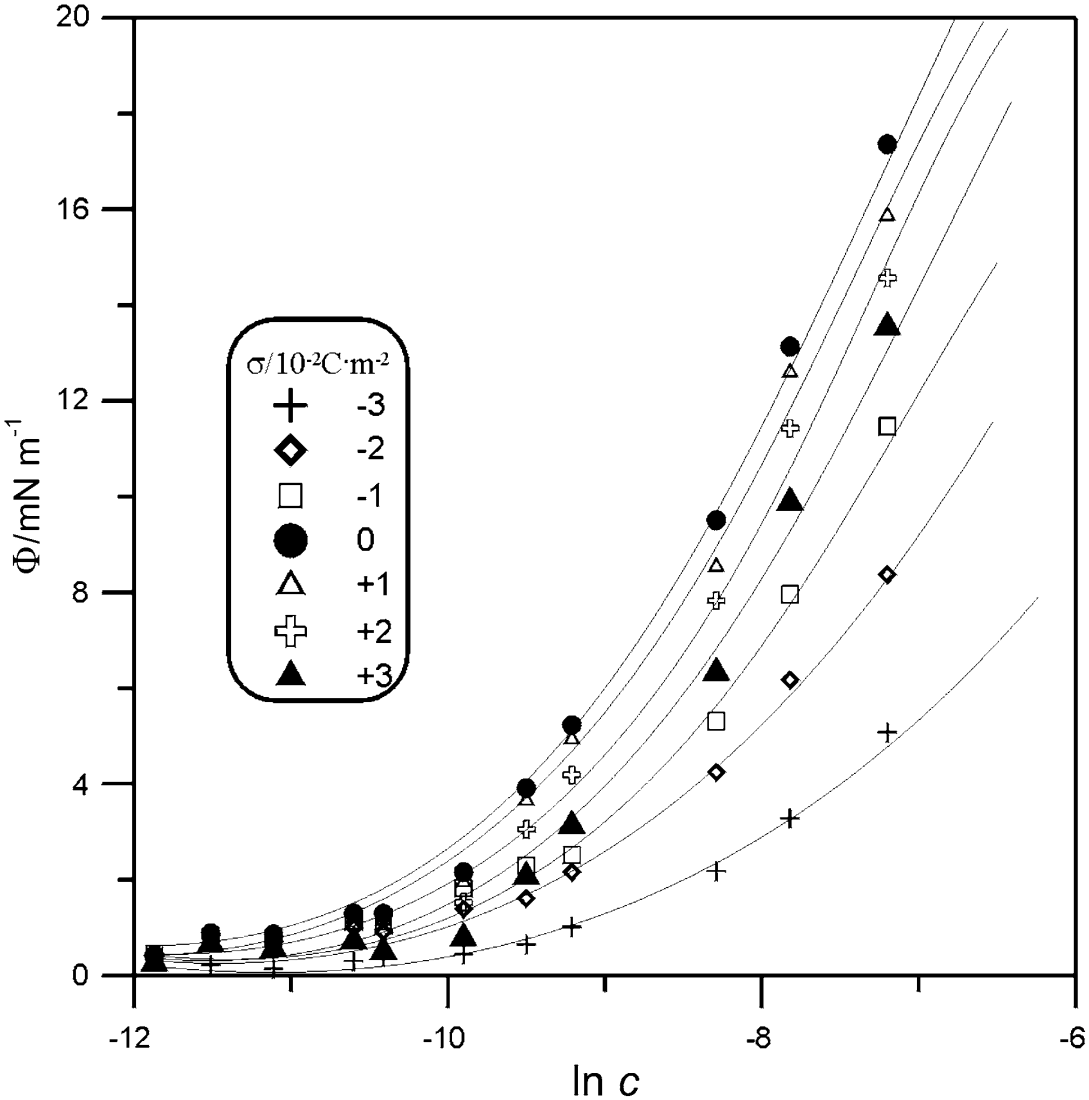
Surface pressure, $\varPhi$, vs. ${\text{C}}_{ 1 0} {\text{H}}_{ 2 1} {\text{SO}}_{ 3}^{ - }$ concentration, ln*c* and the electrode charge, $\sigma$

**Fig. 4:**
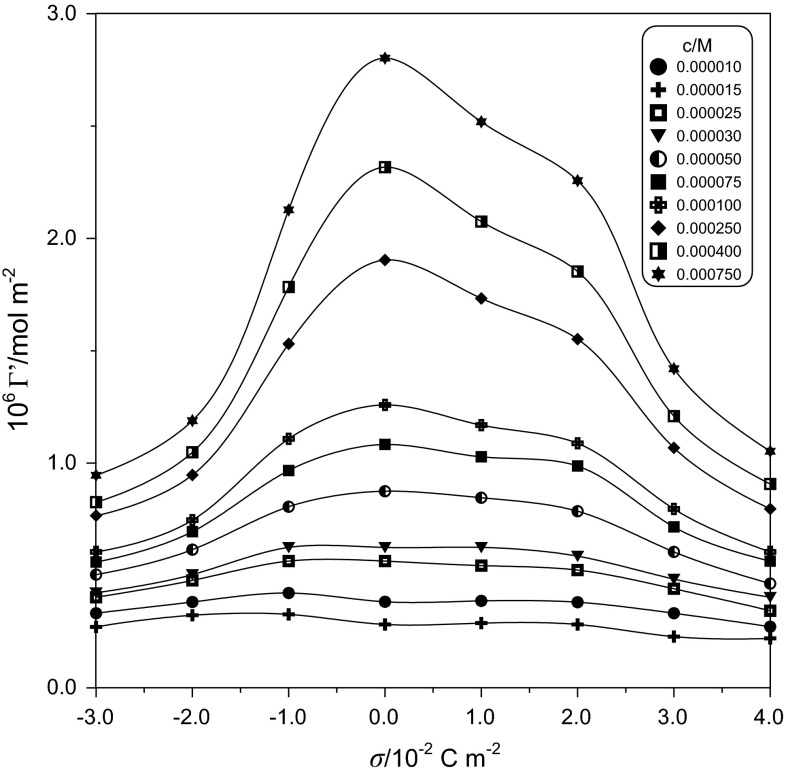
Relative surface excess of ${\text{C}}_{ 1 0} {\text{H}}_{ 2 1} {\text{SO}}_{ 3}^{ - }$ as a function of the electrode charge and the ${\text{C}}_{ 1 0} {\text{H}}_{ 2 1} {\text{SO}}_{ 3}^{ - }$ concentration in the bulk

The values of *E*_z_ in the studied ${\text{C}}_{ 1 0} {\text{H}}_{ 2 1} {\text{SO}}_{ 3}^{ - }$ concentration change slightly from −461 mV for 1 M NaClO_4_ to −458 mV for 7.5 × 10^−4^ M ${\text{C}}_{ 1 0} {\text{H}}_{ 2 1} {\text{SO}}_{ 3}^{ - }$. This result indicates the mechanism of the anionic surfactant absorption on the mercury electrode: through an alkyl located at the surface of mercury and a sulfonic group directed into the solution. At the same time, a significant change in *γ*_z_ is observed from 421 to 404 mN m^−1^. Using integration constants: *E*_z_ and *γ*_z_, the capacity vs. potential data were numerically integrated from the point of *E*_z_. Figure 2 presents the dependence of the electrode charge, *σ*, vs. the electrode potential, *E*. The obtained dependencies allow to determine the maximum adsorption parameters for ${\text{C}}_{ 1 0} {\text{H}}_{ 2 1} {\text{SO}}_{ 3}^{ - }$ on the mercury electrode: *E*_max_ = −467 mV and *σ*_max_ = 0. The data obtained by integration of differential capacity curves were subsequently used to calculate the Parsons’ auxiliary function $\varsigma = \gamma + \sigma E$ [10]. As the adsorption of ${\text{ClO}}_{ 4}^{ - }$ ions was demonstrated earlier [11], the adsorption of ${\text{C}}_{ 1 0} {\text{H}}_{ 2 1} {\text{SO}}_{ 3}^{ - }$ was described using the relative surface excess, *Г′*, which, according to the Gibbs adsorption isotherm, is given by:

1 $$\varGamma^{'} = \frac{1}{RT}\left( {\frac{\partial \varPhi }{\partial \ln c}} \right)_{\sigma }$$

where *c* is the bulk concentration of ${\text{C}}_{ 1 0} {\text{H}}_{ 2 1} {\text{SO}}_{ 3}^{ - }$ and $\varPhi$ is the surface pressure $\varPhi = \Delta \varsigma = \varsigma_{0} - \varsigma$ ($\varsigma_{0}$ and $\varsigma$ are the values of the Parsons’ auxiliary function for the base electrolyte and the solution containing ${\text{C}}_{ 1 0} {\text{H}}_{ 2 1} {\text{SO}}_{ 3}^{ - }$, respectively). Figure 3 presents the values of $\varPhi$ versus ln*c* and $\sigma$. The obtained values of *Г′* are presented in Fig. 4. For lower concentrations of the anion surfactant, a small dependence of *Г′* on the surface charge is observed. For concentrations *c* ≥1 × 10^−4^ M, a clear maximum occurs at $\sigma$ = 0. The shape of the curves in Fig. 4 shows competitive electrostatic interactions between organic molecules and water dipoles [12].

### Adsorption isotherms

The adsorption of ${\text{C}}_{ 1 0} {\text{H}}_{ 2 1} {\text{SO}}_{ 3}^{ - }$ was further analyzed on the basis of constants obtained from the Frumkin and the modified Flory–Huggins [13–16] isotherms. The Frumkin isotherm constants were determined from the equation.

2 $$\beta x = \left[ {{\raise0.7ex\hbox{$\varTheta $} \!\mathord{\left/ {\vphantom {\varTheta {\left( {1 - \varTheta } \right)}}}\right.\kern-0pt} \!\lower0.7ex\hbox{${\left( {1 - \varTheta } \right)}$}}} \right]\exp \left( { - 2A_{F} \varTheta } \right)$$

where *x* is the mole fraction of $${\text{C}}_{ 1 0} {\text{H}}_{ 2 1} {\text{SO}}_{ 3}^{ - }$$ in the solution, *β* is the adsorption coefficient: $$\beta = \exp \left( {{\raise0.7ex\hbox{${ - \Delta G^\circ }$} \!\mathord{\left/ {\vphantom {{ - \Delta G^\circ } {\text{RT}}}}\right.\kern-0pt} \!\lower0.7ex\hbox{${\text{RT}}$}}} \right)$$, ∆*G*° is the standard Gibbs energy of adsorption, *A*_F_ is the interaction parameter, and $$\varTheta$$ is the coverage value $$\left( {\varTheta = {\raise0.7ex\hbox{${\varGamma^{'} }$} \!\mathord{\left/ {\vphantom {{\varGamma^{'} } {\varGamma_{s} }}}\right.\kern-0pt} \!\lower0.7ex\hbox{${\varGamma_{s} }$}}} \right)$$. The surface excess at saturation, $$\varGamma_{s}$$, was estimated by extrapolating the $${\raise0.7ex\hbox{$1$} \!\mathord{\left/ {\vphantom {1 {\varGamma^{'} }}}\right.\kern-0pt} \!\lower0.7ex\hbox{${\varGamma^{'} }$}}{\text{vs}}.{\raise0.7ex\hbox{$1$} \!\mathord{\left/ {\vphantom {1 {c_{{{\text{c}}_{ 1 0} {\text{H}}_{ 2 1} {\text{SO}}_{ 3}^{ - } }} }}}\right.\kern-0pt} \!\lower0.7ex\hbox{${c_{{{\text{c}}_{ 1 0} {\text{H}}_{ 2 1} {\text{SO}}_{ 3}^{ - } }} }$}}$$ lines at different electrode charges to $${\raise0.7ex\hbox{$1$} \!\mathord{\left/ {\vphantom {1 {c_{{{\text{c}}_{ 1 0} {\text{H}}_{ 2 1} {\text{SO}}_{ 3}^{ - } }} }}}\right.\kern-0pt} \!\lower0.7ex\hbox{${c_{{{\text{c}}_{ 1 0} {\text{H}}_{ 2 1} {\text{SO}}_{ 3}^{ - } }} }$}} = 0$$. The $$\varGamma_{s}$$ value obtained this way was 7.14 × 10^−6^ mol m^−2^. The surface occupied by one $${\text{C}}_{ 1 0} {\text{H}}_{ 2 1} {\text{SO}}_{ 3}^{ - }$$ anion, *S* (*S* = 1/*Г*_s_) was 0.233 nm^2^. Such a small *S* value may indicate a perpendicular orientation of the adsorbed anion with its alkyl directed to the surface of the electrode.

**Fig. 5:**
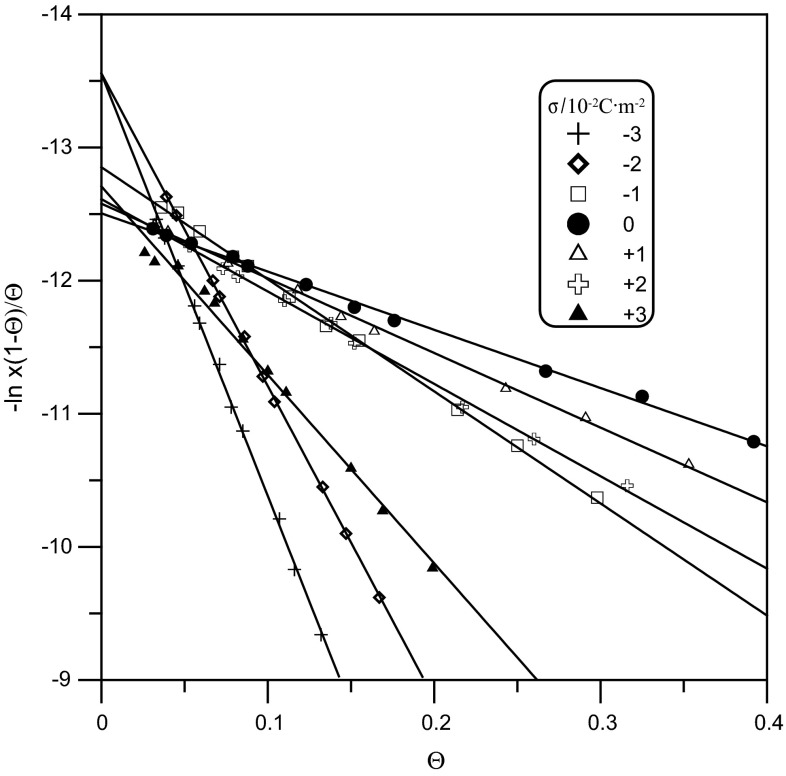
Linear test of the Frumkin isotherm in 1 M NaClO_4_ for different electrode charge values 10^2^ $\sigma$ (C m^−2^)

The values of the *A*_F_ parameter were calculated from the slopes of the lines in the linear test of the Frumkin isotherm and the corresponding $$\Delta G_{F}^{^\circ }$$ values were determined by extrapolation of $$\ln \left[ {\left( {{\raise0.7ex\hbox{${1 - \varTheta }$} \!\mathord{\left/ {\vphantom {{1 - \varTheta } \varTheta }}\right.\kern-0pt} \!\lower0.7ex\hbox{$\varTheta $}}} \right)} \right]$$ vs. $$\varTheta$$ lines to the value of $$\varTheta$$  = 0 (Fig. 5). The obtained values of the Frumkin isotherm constants are presented in Table 1. The lowest value of ∆*G*° is associated with the weakest repulsive interactions. Thus, the maximum values of the relative surface excess for surface electrode charges close to zero are determined by the *A*_F_ parameter, and not the adsorption energy, $$\Delta G_{F}^{^\circ }$$. The adsorption of $${\text{C}}_{ 1 0} {\text{H}}_{ 2 1} {\text{SO}}_{ 3}^{ - }$$ was further analyzed based on $$\Delta G_{H}^{^\circ }$$ and *A*_H_ constants obtained from the modified Flory–Huggins isotherm for a long-range particle–particle interaction:

**Table 1:** The constants of Frumkin (*F*), corrected Flory–Huggins (*H*), and virial (*V*) isotherms for the following system: 1 M NaClO_4_ + ${\text{C}}_{ 1 0} {\text{H}}_{ 2 1} {\text{SO}}_{ 3}^{ - }$; 10^2^ $\sigma$ (C m^−2^); $\Delta G^{o}$ (kJ mol^−1^)

$\sigma$	$\Delta G_{F}^{^\circ }$	−*A*_F_	$\Delta G_{H}^{^\circ }$	−*A*_H_	−$\Delta G_{V}^{^\circ }$	*B*
−3	33.6	16.1	35.0	14.6	112.7	3.3
−2	33.6	11.6	35.0	10.8	112.7	2.5
−1	31.8	4.2	33.4	3.8	111.1	1.0
0	31.1	2.3	32.6	1.7	110.2	0.6
+1	31.1	2.7	32.7	2.3	110.3	0.7
+2	31.2	3.4	32.9	3.0	110.3	0.8
+3	31.5	7.2	33.1	7.2	110.0	1.6
+4	31.8	11.6	33.3	10.8	110.0	2.4

3 $$\beta x = \left[ {{\raise0.7ex\hbox{$\varTheta $} \!\mathord{\left/ {\vphantom {\varTheta {n\left( {1 - \varTheta } \right)^{n} }}}\right.\kern-0pt} \!\lower0.7ex\hbox{${n\left( {1 - \varTheta } \right)^{n} }$}}} \right]\exp \left( { - 2A_{H} \varTheta } \right)$$

where *n* = 1.89 is the number of water molecules replaced by one ${\text{C}}_{ 1 0} {\text{H}}_{ 2 1} {\text{SO}}_{ 3}^{ - }$ anion. In the presented case the projected area for water [15] is 0.123 nm^2^. As ${\text{ClO}}_{ 4}^{ - }$ ions cause the strongest disruption in the water structure [7], the surface of one water molecule was used in calculations, instead of the surface of water clusters. The calculations indicate a slightly higher $\Delta G_{H}^{^\circ }$ values and slightly weaker repulsive interactions, compared to the constants obtained from the Frumkin isotherm. However, the tendencies of changes are similar.

The data obtained from the above-mentioned isotherms were verified using the virial isotherm. The application of virial isotherm does not require knowing the *Г*_s_ value. The virial isotherm equation is:

4 $$\ln \beta c = \ln \varGamma^{'} + 2B\varGamma^{'}$$

where *B* is the two-dimensional (2D) second virial coefficient which was calculated from the slopes of the lines on the linear test of the virial isotherm: $$\log \frac{{\varGamma^{'} }}{c}{\text{vs}}.\;\varGamma^{'}$$. The $$\Delta G_{V}^{^\circ }$$ values were obtained from the intercepts of these lines with the axis $$\log \left( {{\raise0.7ex\hbox{${\varGamma^{'} }$} \!\mathord{\left/ {\vphantom {{\varGamma^{'} } c}}\right.\kern-0pt} \!\lower0.7ex\hbox{$c$}}} \right)$$ using the standard state of 1 mol dm^−3^ in the bulk solution and 1 mol cm^−2^ on the electrode surface. The obtained constants values are presented in Table 1. The calculated values of $$\Delta G_{V}^{^\circ }$$ and *B* confirm the tendencies of changes determined based on the Frumkin and Flory–Huggins isotherm. It is noteworthy that the values of ∆*G*° and interaction constants are close to respective values obtained for tetramethylthiourea [17] during chemical adsorption on a mercury electrode. The lower values obtained for *Г′* of tetramethylthiourea result from much lower concentrations of $${\text{C}}_{ 1 0} {\text{H}}_{ 2 1} {\text{SO}}_{ 3}^{ - }$$. The ∆*G*° values presented in Table 1 confirm strong physical adsorption of the studied surfactant on the mercury electrode caused by the alkyl. The repulsive interaction between $$-{\text{SO}}_{ 3}^{ - }$$ groups increases very significantly with growing distance from the electrode zero charge. This effect is more clear for $$\sigma$$ < 0, compared to $$\sigma$$ > 0. Thus, the negative surface charge of the electrode influences the reorientation of the adsorbed $${\text{C}}_{ 1 0} {\text{H}}_{ 2 1} {\text{SO}}_{ 3}^{ - }$$ anions.

## Conclusions

The presented results led us to the following conclusions:

1. Low concentrations of the anionic surfactant (below cmc) causes slight changes in the zero charge potential, *E*_z_, and the surface tension at this potential, *γ*_z_.
2. We found the character of ${\text{C}}_{ 1 0} {\text{H}}_{ 2 1} {\text{SO}}_{ 3}^{ - }$ adsorption to be physical, which is reflected by the ability to determine the parameters of maximum adsorption: *E*_max_ and $\sigma$_max_, and the bell-shaped $\varGamma^{'} = f\left( \sigma \right)$ curve.
3. The determined values of the standard Gibbs energy of adsorption ∆*G*° indicate strong adsorption of the studied surfactant, comparable to chemical adsorption.
4. The values of relative surface excess, *Г* ^′^, are clearly related to the interaction constant *A* or *B*, and not the energy of adsorption.
5. The negative surface charges on the electrode have a stronger impact on the reorientation of adsorbed ${\text{C}}_{ 1 0} {\text{H}}_{ 2 1} {\text{SO}}_{ 3}^{ - }$ anions, compared to positive charges.

## Experimental

The experiments were performed in a three-electrode system with a dropping mercury electrode as the working electrode, Ag/AgCl with saturated sodium chloride as the reference electrode, and a platinum spiral as the counter electrode. The differential capacity of the double layer, *C*, was measured using the ac impedance technique using an Autolab frequency response analyzer (Eco Chemie, The Netherlands). The measurements were carried out at several frequencies in the range of 400–2,000 Hz with an amplitude of 5 mV. The equilibrium capacities were obtained by extrapolation of the measured capacity vs. square root of the frequency curve to zero frequency.

The potential of zero charge, *E*_z_, was measured using a streaming electrode [8]. The interfacial tension, *γ*_z_, at *E*_z_ was measured using the maximum-bubble pressure method according to Schiffrin [9]. The charge density and the surface tension for the studied system (1 M NaClO_4_ and an increasing concentration of ${\text{C}}_{ 1 0} {\text{H}}_{ 2 1} {\text{SO}}_{ 3}^{ - }$ from 7.5 × 10^−6^–7.5 × 10^−4^ M) were obtained through back integration of differential capacity–potential dependencies.

Analytical grade C_10_H_21_SO_3_Na and NaClO_4_ (Fluka) were used without any further purification. Water and mercury were double distilled before use. The solutions were deaerated by passing high-purity nitrogen over the solutions during the measurements, which were carried out at 298 ± 0.1 K.
